# GOOD FOR HEALTH, BAD FOR SUBJECTIVE WELL-BEING: EFFECT OF CONTINUING WORK ON JAPANESE OLDER ADULTS

**DOI:** 10.1093/geroni/igad104.1063

**Published:** 2023-12-21

**Authors:** Keiko Katagiri

**Affiliations:** Kobe University, Kobe, Hyogo, Japan

## Abstract

Recently, the labor force rate of Japanese older adults has been increasing. The white paper of Japan’s Cabinet Office shows that half of the people in their late 60s are employed in 2022. Previous literature has indicated that work has positive effects on health and cognitive functions; however, the work environment is not age-friendly. Most companies set the mandatory retirement age at 60, after which people must work under a worse labor contract. This study aims to explore the effect of work on Japanese older adults’ subjective well-being and to examine whether social participation and civic activity can buffer work’s negative effects. Random sampling survey was conducted in 2019 on people living in the Tokyo and Hyogo prefectures. The response rate was 43.0% (N = 1,063, aged 50–74). Analyses of covariance were conducted on well-being, depression, anxiety, loneliness, and life satisfaction. We found that work has negative effects on anxiety and life satisfaction, with civic activity moderating the negative effect on life satisfaction. Gender differences were observed regarding anxiety and loneliness. Women showed higher anxiety, and men showed higher loneliness. Regarding age difference, people in their late 60s exhibited the lowest depression and those in their late 50s higher anxiety. It is argued that Japan’s third agers live in more difficult work circumstances than ever before, and it is thus necessary to explore age-friendly labor conditions for older adults.

